# Association between spicy foods consumption and cardiovascular disease risk factors: Guangzhou Biobank Cohort Study

**DOI:** 10.1186/s12889-022-13697-6

**Published:** 2022-06-30

**Authors:** Yu Zhang, Zi Long Lu, Wei Sen Zhang, Ya Li Jin, Tong Zhu, Lin Xu

**Affiliations:** 1 School of Public Health, Sun Yat-sen University, 74 Zhongshan 2nd Road, Guangzhou, Guangdong Province China; 2 Guangzhou Twelfth People’s Hospital, Guangzhou, 510620 China; 3 School of Public Health, the University of Hong Kong, Hong Kong, Hong Kong

**Keywords:** Spicy foods, Cardiovascular disease, Diabetes, Obesity, Risk factor

## Abstract

**Background:**

Evidence regarding the association of spicy foods intake with cardiovascular disease (CVD) risk factors was less clear, especially in those with diabetes. We hereby examined the association of spicy foods intake with CVD risk factors in older Chinese.

**Methods:**

Thirty thousand three hundred twenty-five participants (72.34% women) aged 50+ years were recruited in the Guangzhou Biobank Cohort Study from 2003 to 2008. Information of spicy foods intake and disease history was collected by face-to-face interview. CVD risk factors were measured and treated as continuous variables. Diabetes was defined by a fasting plasma glucose (FPG) ≥7.0 mmol/L and/or self-reported physician-diagnosed diabetes.

**Results:**

Of 30,325 participants, 12.9% consumed spicy foods regularly. After adjusting for multiple confounders, participants who consumed spicy foods of 5–7 days/week, versus none, had higher body mass index (1.18, 95% confidence interval (CI) 0.95 to 1.42 kg/m^2^), waist circumference (2.80, 95% CI 2.18–3.41 cm), waist-to-hip ratio (0.010, 95% CI 0.006 to 0.015), systolic blood pressure (2.44, 95% CI 0.92 to 3.97 mmHg), diastolic blood pressure (1.94, 95% CI 1.14 to 2.73 mmHg), FPG (0.310, 95% CI 0.188 to 0.432 mmol/L), triglycerides (0.185, 95% CI 0.096 to 0.273 mmol/L), and lower high-`density lipoprotein cholesterol (− 0.040, 95% CI − 0.069 to − 0.012 mmol/L). Similar results were found for the associations of spicy foods strength with CVD risk factors. The results attenuated slightly but not substantially across diabetes groups.

**Conclusions:**

Our study showed that higher frequency and strength of spicy foods intake were associated with unfavorable cardiovascular disease risk profile in older people, and such associations did not vary by diabetes status. Whether the results were causal needs to be determined in further studies.

**Supplementary Information:**

The online version contains supplementary material available at 10.1186/s12889-022-13697-6.

## Background

Chronic diseases such as cardiovascular disease, cancer and diabetes have become major causes of death globally and in China [1, 2], among which cardiovascular disease accounts for 40% of the total death [3]. Identifying modifiable CVD risk factors is of great significance in terms of setting up primary prevention strategies. Unhealthy diet has been considered a key modifiable risk factor for many chronic diseases [4–7]. Lifestyle changes, especially modification in dietary pattern, are recommended as part of prevention and control strategies for metabolic disorders such as dyslipidemia and insulin resistance [8].

Spices have a long history of use for coloring, flavoring and preserving food, as well as for medicinal purposes worldwide [9], and they are considered to have multiple beneficial effects on human health. For example, the China Kadoorie Biobank (CKB) study shows that high levels of spicy food intake are associated with a lower risk of certain gastrointestinal cancers in Chinese adults [10]. Previous studies describing the association between spicy foods intake and the risk of CVD showed inconsistent results [11–14]. Some studies found that spicy food consumption was associated with lower risks of hypertension [15, 16], obesity [17] and diabetes [18], and lower levels of lipids [8, 19]. However, there is also evidence that spicy foods intake was positively associated with obesity indices such as body mass index (BMI) and waist circumference (WC) in Chinese [20–22]. The discrepancies could be partly explained by with [17, 20, 22] or without [21] adjustment for total energy intake.

Furthermore, all previous studies did not account for moderating effect of diabetes. As people with diabetes may have changed their dietary patterns, which, if not accounted for, may lead to biased estimates, we also aimed to explore whether the association varied by diabetes. Therefore, we conducted this study to explore the dose-response patterns of the association between spicy foods intake and CVD risk factors in older Chinese overall and by diabetes status.

## Methods

### Study population

All participants of the Guangzhou Biobank Cohort Study (GBCS) were recruited from 2003 to 2008. The Guangzhou Medical Ethics Committee of the Chinese Medical Association approved the study and all participants gave written, informed consent before participation.

Details of the GBCS have been described previously [23]. Briefly, the GBCS is a 3-way collaboration among the Guangzhou Twelfth People’s Hospital, the University of Hong Kong in China, and the University of Birmingham in the UK. Participants were recruited from The Guangzhou Health and Happiness Association for the Respectable Elders (GHHARE), a community social and welfare organization. Membership is open to permanent residents of Guangzhou aged 50 years or older for a nominal fee of 4 CNY (about 50 US cents) per month [24]. GHHARE included about 7% of Guangzhou residents in this age group, with branches in all 10 districts of Guangzhou, the capital city of Guangdong Province in southern China [25].

Information of demographic characteristics, lifestyle, and personal medical history were collected at recruitment by face-to-face interview by trained nurses using a computer-assisted questionnaire. Anthropometric measurements were also measured by trained nurses using the standard protocol. Physical activity was categorized into inactive, minimally active, and active based on the short version of the International Physical Activity Questionnaire (IPAQ). Fasting blood samples are obtained from all participants after overnight fasted, and glucose was determined automatically in the hospital laboratory [23].

### Exposures

Information of the frequency, strength and duration of consumption of spicy food were collected by self-report in a face-to-face interview. Spicy foods refer to foods with spices for flavoring including chilli sauce, chilli oil, dried capsicum, fresh capsicum and others. Frequency of intake was categorized into four groups: never (< 1 day/week), sometimes (1–2 days/week), often (3–4 days/week), usually (≥5 days/week). Degree of pungency was categorized into the following four groups: non-eating, mild pungency, moderate pungency, strong pungency.

### Outcomes

The study outcomes were CVD risk factors including systolic and diastolic blood pressure (SBP and DBP), lipids (low-density lipoprotein (LDL)-cholesterol, high-density lipoprotein (HDL)-cholesterol, triglycerides and total cholesterol), fasting plasma glucose, and adiposity measures (body mass index (BMI), waist circumference (WC) and waist-to-hip ratio). BMI was calculated by weight in kilograms divided by squared height in meters. All participants were invited to come to a research center in the Guangzhou Twelfth People’s Hospital after an overnight fast, where full-time trained nurses and technicians carried out physical examinations and interviews following standardized procedures. Biochemical tests were performed in a central laboratory of the hospital with good quality control [23].

### Potential confounders or moderators

Lifestyle and sociodemographic factors that were associated with both spicy foods consumption and the risk of cardiovascular disease were included as covariates. Potential confounders included sex, age, occupation, household income, education， physical activity, drinking status, smoking status and energy intake per day. Education was classified as primary school or below, middle school, and college or above. Household income was classified into < 10,000, 10,000-29,999, 30,000-49,999, ≥50,000 Yuan/year and don’t know. Occupation was classified into manual, non-manual and others. Alcohol use and smoking status were classified into never, former, and current users. Detailed diet (semi-quantitative food frequency) was obtained by using a 300-item food frequency questionnaire, as reported in our previous papers [26, 27]. Energy intake per day was calculated by total amount of food consumed last week. Diabetes was defined by a fasting plasma glucose ≥ 7.0 mmol/L, use of anti-diabetes medications, and/or self-reported physician-diagnosed diabetes [28].

### Statistical analysis

Chi-square tests or analysis or variance were used to compare participants’ characteristics by spicy foods consumption. Multivariable linear regression models were used to assess the association of spicy foods consumption with CVD risk factors. We also tested for interactions between frequency of spicy foods intake and diabetes, and between frequency and pungency of spicy foods intake in terms of their associations with CVD risk factors. A significant interaction was determined if significant improvement in model fitness after including the interaction term could be observed using likelihood ratio test, indicated by a reduction in Akaike information criterion (AIC) value. We found suggestive evidence for effect modification for the interactions with diabetes (P for interactions from 0.04 to 0.95) but not for interactions with pungency (*P* for interactions from 0.23 to 0.92). Therefore, subgroup analysis by diabetes was performed to further explore whether the association varied by diabetes. All statistical analyses were done with Stata version 16.0 (STATA Corp LP), and all tests were two-sided with a significance level of 0.05.

## Results

Table 1 shows that 87.1% of participants did not eat any spicy foods and 3.89% ate frequently. Compared with participants who never ate spicy foods, those who consumed spicy foods with a higher frequency or pungency were younger, had a higher proportion of men, current smokers and alcohol users, and had higher levels of BMI, WC and WHR (all *P* values for trend < 0.001). However, no association between physical activity and spicy food consumption was found.

**Table 1:** Sample characteristics by frequency and strength of spicy food consumption

**Frequency of spicy foods consumption**						
	**Never(<1 days/wk.)**	**Sometimes (1-2 days/wk.)**	**Often (3-4 days/wk.)**	**Usually (≥5 days/wk.)**	**Total**	***P*** **value**
Number	26,413	1728	1005	1179	30,325	
Women (%)	72.66	71.82	69.05	68.87	72.34	0.003
Age (y)	62.16 ± 7.16	60.78 ± 6.67	60.85 ± 6.50	61.91 ± 6.95	62.02 ± 7.12	< 0.001
BMI (kg/m^2^)	23.67 ± 3.30	24.27 ± 3.32	24.48 ± 3.36	24.85 ± 3.33	23.77 ± 3.32	< 0.001
WHR	0.867 ± 0.068	0.867 ± 0.067	0.874 ± 0.066	0.878 ± 0.067	0.868 ± 0.068	< 0.001
Waist (cm)	78.56 ± 8.99	79.56 ± 8.87	80.26 ± 8.83	81.46 ± 9.14	78.79 ± 9.01	< 0.001
Education (%)						< 0.001
≤ Primary	44.35	32.89	33.07	34.44	42.94	
Middle School	47.39	54.95	53.89	49.62	48.13	
≥ College or above	8.26	12.16	12.95	15.95	8.93	
Household income (Yuan/year)						< 0.001
< 10,000	6.04	3.82	3.39	3.48	5.73	
10,000-29,999	32.74	28.97	27.89	31.01	32.29	
30,000-49,999	20.92	23.87	23.31	21.75	21.20	
≥ 50,000	16.14	22.54	21.41	22.85	16.94	
Don’t know	24.15	20.80	24.00	20.90	23.83	
Smoking history (%)						< 0.001
Never	81.21	79.88	76.52	75.45	80.76	
Ex-smoker	9.14	7.77	10.15	11.13	9.17	
Current smoker	9.65	12.35	13.33	13.42	10.07	
Drinking status (%)						< 0.001
Never	74.22	58.49	60.64	60.65	72.35	
Ex-drinker	3.45	4.10	4.90	3.58	3.54	
Current drinker	22.33	37.41	34.47	35.78	24.11	
Occupation (%)						< 0.001
Manual	62.30	55.67	51.85	51.28	61.15	
Non-manual	22.79	29.01	29.17	33.22	23.76	
Other	14.91	15.32	18.98	15.50	15.09	
Physical activities (%)						0.332
Inactive	8.14	7.64	7.36	7.97	8.08	
Minimally active	40.95	42.65	42.69	38.59	41.01	
Active	50.91	49.71	49.95	53.44	50.91	
Daily energy intake(kcal/d)						< 0.001
Median	1774.72	1863.36	1883.67	1864.07	1787.86	
Interquartile range	(1452.79,2131.63)	(1495.88,2251.08)	(1560.90,2279.51)	(1473.05,2273.09)	(1458.57,2151.64)	
Daily carbohydrate intake(kcal/d)						< 0.001
Median	252.07	259.77	270.72	263.92	253.65	
Interquartile range	(199.21,310.50)	(201.49, 325.29)	(214.45, 327.98)	(199.1, 329.61)	(199.89,312.83)	
Daily protein intake(kcal/d)						< 0.001
Median	69.08	73.86	72.76	71.77	69.63	
Interquartile range	(56.03,84.63)	(59.63,89.04)	(58.50,87.70)	(57.74,89.75)	(56.37,85.30)	
Daily fat intake(kcal/d)						< 0.001
Median	55.12	59.23	60.17	58.85	55.69	
Interquartile range	(41.03,70.65)	(44.74,74.93)	(44.72,77.62)	(43.55, 75.62)	(41.45,71.34)	
**Strength of spicy foods consumption**						
	**Never**	**Mild**	**Moderate**	**Strong**	**Total**	***P*** **value**
Number	26,413	2407	1121	380	30,321	
Women (%)	72.66	73.74	67.26	56.58	72.34	< 0.001
Age (y)	62.16 ± 7.16	61.39 ± 6.72	60.77 ± 6.66	60.50 ± 6.89	62.02 ± 7.12	< 0.001
BMI (kg/m^2^)	23.67 ± 3.30	24.37 ± 3.29	24.66 ± 3.44	24.86 ± 3.35	23.77 ± 3.32	< 0.001
WHR	0.867 ± 0.068	0.872 ± 0.067	0.873 ± 0.067	0.881 ± 0.065	0.868 ± 0.068	< 0.001
WC (cm)	78.56 ± 8.99	79.98 ± 8.82	80.48 ± 9.08	81.92 ± 9.44	78.79 ± 9.01	< 0.001
Education (%)						< 0.001
≤ Primary	44.35	34.51	31.31	32.63	42.94	
Middle School	47.39	52.06	54.24	56.32	48.13	
≥ College or above	8.26	13.43	14.45	11.05	8.93	
Household income (Yuan/year)						< 0.001
< 10,000	6.04	3.95	3.13	2.63	5.73	
10,000-29,999	32.74	29.52	28.71	29.74	32.30	
30,000-49,999	20.92	22.49	25.31	20.53	21.21	
≥ 50,000	16.14	22.16	22.09	24.47	16.95	
Don’t know	24.15	21.87	20.75	22.63	23.83	
Smoking history (%)						< 0.001
Never	81.22	80.95	75.56	62.96	80.76	
Ex-smoker	9.14	9.23	9.10	11.38	9.17	
Current smoker	9.65	9.82	15.34	25.66	10.07	
Drinking status (%)						< 0.001
Never	74.22	62.29	56.74	51.59	72.35	
Ex-drinker	3.45	3.89	4.32	5.29	3.54	
Current drinker	22.33	33.82	38.94	43.12	24.11	
Occupation (%)						< 0.001
Manual	62.30	54.02	52.10	52.65	61.14	
Non-manual	22.79	28.96	32.05	33.86	23.76	
Other	14.91	17.02	15.85	13.49	15.09	
Physical activities (%)						0.504
Inactive	8.14	7.81	6.96	8.95	8.08	
Minimally active	40.95	40.47	43.53	41.58	41.01	
Active	50.91	51.72	49.51	49.47	50.90	
Daily energy intake(kcal/d)						< 0.001
Median	1774.72	1883.46	1815.78	1911.14	1787.86	
Interquartile range	(1452.79,2131.63)	(1538.36,2268.63)	(1452.68,2223.51)	(1521.69,2315.07)	(1458.58,2151.63)	
Daily carbohydrate intake(kcal/d)						< 0.001
Median	252.07	270.05	252.51	265.72	253.65	
Interquartile range	(199.21,310.50)	(208.29, 330.34)	(191.81, 318.42)	(201.28, 333.47)	(199.89,312.83)	
Daily protein intake(kcal/d)						< 0.001
Median	69.08	73.06	72.65	74.25	69.63	
Interquartile range	(56.03,84.63)	(58.92,88.05)	(57.39, 88.73)	(58.51,94.48)	(56.37,85.30)	
Daily fat intake(kcal/d)						< 0.001
Median	55.12	59.23	58.83	60.77	55.69	
Interquartile range	(41.03,70.65)	(44.48, 76.43)	(44.79,73.44)	(47.78,77.36)	(41.45,71.34)	

Data were expressed as means ± SDs or medians (IQRs) for continuous variables and percentages (%) for categorical variables

Table 2 shows that higher frequency of spicy foods intake was associated with higher levels of BMI, WC, WHR, DBP, fasting plasma glucose and TG, and lower levels of HDL-C in a dose-response manner (all *P* values for trend < 0.001). After adjusting for age, sex, education, household income, occupation, physical activity, drinking, smoking and daily energy intake (model 2), compared with the lowest frequency group, participants with 5–7 days/week of spicy foods intake had higher levels of BMI (1.18, 95% confidence interval (CI) 0.95–1.42 kg/m^2^), WC (2.80, 95% CI 2.18–3.41 cm), WHR (0.010, 95% CI 0.006–0.015), SBP (2.44, 95% CI 0.92–3.97 mmHg), DBP (1.94, 95% CI 1.14–2.73 mmHg), fasting plasma glucose (0.310, 95% CI 0.188–0.432 mmol/L) and triglycerides (0.185, 95% CI 0.096–0.273 mmol/L), and lower levels of HDL-C (− 0.040, 95% CI -0.069--0.012 mmol/L). Furthermore, no significant association was observed between spicy foods intake and levels of LDL-cholesterol or TC.

**Table 2:** Associations between frequency of spicy food consumption and CVD risk factors

	Frequency of spicy foods consumption; β and 95% confidence interval					
	Never(<1 day/wk.)	Sometimes (<2 days/wk.)	Often (3-4 days/wk.)	Usually (≥5 days/wk.)	Adjusted ***R-***squared	***P for trend***
**No. of participants**	26,413	1728	1005	1179		
**BMI (kg/m**^**2**^**)**	23.67 ± 3.30	24.27 ± 3.32	24.48 ± 3.36	24.85 ± 3.33		< 0.001
Crude model	Ref	0.61(0.45,0.77)	0.81(0.61,1.02)	1.18(0.99,1.38)	0.0077	< 0.001
Model 1	Ref	0.62(0.46,0.79)	0.85(0.64,1.06)	1.24(1.05,1.44)	0.0226	< 0.001
Model 2	Ref	0.62(0.43,0.81)	0.87(0.62,1.12)	1.18(0.95,1.42)	0.0220	< 0.001
Model 3	Ref	0.62(0.43,0.81)	0.88(0.63,1.13)	1.19(0.95,1.42)	0.0232	< 0.001
**WC (cm)**	78.56 ± 8.99	79.56 ± 8.87	80.26 ± 8.83	81.46 ± 9.14		< 0.001
Crude model	Ref	0.99(0.55,1.43)	1.70(1.13,2.26)	2.90(2.37,3.42)	0.0052	< 0.001
Model 1	Ref	1.41(0.99,1.84)	1.97(1.42,2.52)	2.99(2.48,3.50)	0.0774	< 0.001
Model 2	Ref	1.39(0.89,1.89)	1.89(1.25,2.54)	2.80(2.18,3.41)	0.0793	< 0.001
Model 3	Ref	1.39(0.89,1.89)	1.91(1.26,2.56)	2.81(2.20,3.43)	0.0806	< 0.001
**WHR**	0.867 ± 0.068	0.867 ± 0.067	0.874 ± 0.066	0.878 ± 0.067		< 0.001
Crude model	Ref	0.001(−0.003,0.004)	0.007(0.002,0.011)	0.011(0.007,0.015)	0.0012	< 0.001
Model 1	Ref	0.004(0.001,0.008)	0.009(0.005,0.013)	0.012(0.008,0.016)	0.1434	< 0.001
Model 2	Ref	0.005(0.001,0.008)	0.009(0.004,0.014)	0.010(0.006,0.015)	0.1404	< 0.001
Model 3	Ref	0.005(0.001,0.008)	0.009(0.004,0.014)	0.010(0.006,0.015)	0.1408	< 0.001
**SBP (mmHg)**	130.34 ± 22.13	129.60 ± 22.17	130.89 ± 22.30	131.87 ± 22.36		0.044
Crude model	Ref	−0.74(−1.82,0.34)	0.55(− 0.84,1.95)	1.53(0.24,2.83)	0.0002	0.063
Model 1	Ref	1.27(0.22,2.31)	2.18(0.84,3.52)	2.67(1.42,3.91)	0.0944	< 0.001
Model 2	Ref	1.31(0.06,2.55)	2.31(0.70,3.92)	2.44(0.92,3.97)	0.0778	< 0.001
Model 3	Ref	1.32(0.07,2.56)	2.34(0.73,3.95)	2.47(0.95,4.00)	0.0783	< 0.001
**DBP (mmHg)**	73.47 ± 11.22	74.11 ± 11.19	74.62 ± 11.59	75.24 ± 11.50		< 0.001
Crude model	Ref	0.63(0.09,1.18)	1.15(0.44,1.86)	1.77(1.11,2.43)	0.0012	< 0.001
Model 1	Ref	0.92(0.37,1.47)	1.26(0.55,1.96)	2.02(1.37,2.67)	0.0330	< 0.001
Model 2	Ref	0.79(0.14,1.45)	1.18(0.34,2.03)	1.94(1.14,2.73)	0.0307	< 0.001
Model 3	Ref	0.80(0.15,1.46)	1.19(0.34,2.03)	1.96(1.16,2.76)	0.0318	< 0.001
**Glucose (mmol/L)**	5.73 ± 1.63	5.82 ± 1.85	5.85 ± 1.68	6.01 ± 2.01		< 0.001
Crude model	Ref	0.093(0.012,0.175)	0.117(0.012,0.222)	0.278(0.180,0.375)	0.0012	< 0.001
Model 1	Ref	0.136(0.054,0.219)	0.173(0.068,0.278)	0.310(0.212,0.407)	0.0154	< 0.001
Model 2	Ref	0.150(0.050,0.250)	0.187(0.058,1.316)	0.310(0.188,0.432)	0.0176	< 0.001
Model 3	Ref	0.147(0.047,0.246)	0.193(0.063,0.322)	0.310(0.188,0.432)	0.0194	< 0.001
**HDL-cholesterol (mmol/L)**	1.66 ± 0.41	1.64 ± 0.41	1.63 ± 0.41	1.61 ± 0.39		< 0.001
Crude model	Ref	−0.019 (− 0.039,0.000)	−0.031(− 0.056, − 0.005)	− 0.051(− 0.074, − 0.027)	0.0007	< 0.001
Model 1	Ref	− 0.018(− 0.037,0.002)	− 0.022(− 0.047,0.003)	−0.045(− 0.068,-0.022)	0.0495	< 0.001
Model 2	Ref	−0.012(− 0.035,0.011)	−0.006(− 0.036,0.025)	−0.040(− 0.069,-0.012)	0.0500	0.008
Model 3	Ref	−0.013(− 0.036,0.010)	−0.004(− 0.034,0.026)	−0.041(− 0.069,-0.013)	0.0561	0.007
**LDL-cholesterol (mmol/L)**	3.26 ± 0.71	3.31 ± 0.71	3.25 ± 0.68	3.24 ± 0.71		0.022
Crude model	Ref	0.050 (0.015,0.084)	−0.010 (− 0.054,0.035)	− 0.022 (− 0.063,0.019)	0.0002	0.783
Model 1	Ref	0.019(−0.015,0.054)	− 0.017(− 0.061,0.027)	−0.028(− 0.068,0.013)	0.0481	0.261
Model 2	Ref	0.067(0.028,0.106)	0.005(−0.046,0.056)	−0.029(− 0.077,0.019)	0.0610	0.996
Model 3	Ref	0.065(0.026,0.104)	0.006(−0.044,0.057)	−0.031(− 0.079,0.017)	0.0691	0.961
**TG (mmol/L)**	1.66 ± 1.22	1.74 ± 1.51	1.77 ± 1.45	1.83 ± 1.49		< 0.001
Crude model	Ref	0.075 (0.014,0.137)	0.102 (0.023,0.182)	0.161 (0.087,0.234)	0.0008	< 0.001
Model 1	Ref	0.077(0.015,0.140)	0.107(0.027,0.187)	0.171(0.097,0.245)	0.0027	< 0.001
Model 2	Ref	0.066(−0.007,0.138)	0.051(−0.043,0.145)	0.185(0.096,0.273)	0.0032	< 0.001
Model 3	Ref	0.066(−0.007,0.138)	0.053(−0.041,0.147)	0.186(0.097,0.275)	0.0034	< 0.001
**TC (mmol/L)**	5.92 ± 1.14	5.99 ± 1.16	5.91 ± 1.09	5.88 ± 1.12		0.054
Crude model	Ref	0.066 (0.011,0.122)	−0.013 (−0.085,0.059)	−0.044 (− 0.111,0.022)	0.0002	0.566
Model 1	Ref	0.056(0.001,0.112)	0.000(−0.071,0.071)	−0.032(− 0.099,0.034)	0.0354	0.799
Model 2	Ref	0.064(−0.003,0.130)	−0.013(− 0.099,0.074)	−0.071(− 0.152,0.011)	0.0377	0.315
Model 3	Ref	0.060(−0.007,0.126)	−0.009(− 0.095,0.077)	−0.073(− 0.154,0.008)	0.0444	0.300

Data were expressed as β coefficients and 95% confidence interval, or mean ± standard deviation

Model 1: adjusted for sex, age, education, household income, occupation, physical activity, drinking status, smoking status

Model 2: additionally adjusted for daily energy intake

Model 3: additionally adjusted for daily carbohydrate, fat and protein intake

Table 3 shows that the pungency of spicy foods was also positively associated with all adiposity measures, DBP, fasting plasma glucose and TG after similar adjustment (all *P* values for trend < 0.001). Additionally adjusting for three variables (i.e., daily carbohydrate, protein and fat intake) showed similar association of frequency and strength of spicy foods consumption with levels of BMI, WC, WHR, DBP, fasting plasma glucose TG and HDL-cholesterol in a dose-response manner (Tables 2 and 3 and Supplementary Figs. 1 and 2).

**Table 3:** Associations between strength of spicy food consumption and CVD risk factors

	Strength of spicy foods consumption; β and 95% confidence interval					
	Never	Mild	Moderate	Strong	Adjusted ***R-***squared	***P for trend***
**No. of participants**	26,413	2407	1121	380		
**BMI (kg/m**^**2**^**)**	23.67 ± 3.30	24.37 ± 3.29	24.66 ± 3.44	24.86 ± 3.35		< 0.001
Crude model	Ref	0.70(0.57,0.84)	1.00(0.80,1.19)	1.19(0.86,1.53)	0.0074	< 0.001
Model 1	Ref	0.72(0.58,0.86)	1.04(0.84,1.23)	1.35(1.01,1.68)	0.0223	< 0.001
Model 2	Ref	0.68(0.51,0.84)	1.07(0.83,1.31)	1.38(0.97,1.80)	0.0221	< 0.001
Model 3	Ref	0.67(0.51,0.84)	1.07(0.84,1.31)	1.41(1.00,1.83)	0.0232	< 0.001
**WC (cm)**	78.56 ± 8.99	79.98 ± 8.82	80.48 ± 9.08	81.92 ± 9.44		< 0.001
Crude model	Ref	1.42(1.04,1.80)	1.91(1.38,2.45)	3.36(2.45,4.27)	0.0770	< 0.001
Model 1	Ref	1.77(1.40,2.13)	2.18(1.65,2.70)	3.40(2.52,4.29)	0.0765	< 0.001
Model 2	Ref	1.61(1.18,2.04)	2.10(1.48,2.72)	3.62(2.54,4.70)	0.0792	< 0.001
Model 3	Ref	1.61(1.18,2.04)	2.13(1.51,2.75)	3.69(2.61,4.77)	0.0805	< 0.001
**WHR**	0.867 ± 0.068	0.871 ± 0.067	0.873 ± 0.067	0.881 ± 0.065		< 0.001
Crude model	Ref	0.004(0.001,0.007)	0.006(0.002,0.010)	0.014(0.007,0.021)	0.0009	< 0.001
Model 1	Ref	0.007(0.005,0.010)	0.008(0.004,0.012)	0.012(0.006,0.019)	0.1431	< 0.001
Model 2	Ref	0.007(0.003,0.010)	0.007(0.003,0.011)	0.015(0.007,0.023)	0.1404	< 0.001
Model 3	Ref	0.007(0.003,0.010)	0.007(0.003,0.012)	0.015(0.007,0.023)	0.1407	< 0.001
**SBP (mmHg)**	130.34 ± 22.13	131.08 ± 21.85	129.48 ± 22.91	131.19 ± 22.98		0.183
Crude model	Ref	0.74(−0.19,1.67)	−0.86(−2.19,0.47)	0.86(−1.39,3.10)	0.0001	0.772
Model 1	Ref	2.19(1.29,3.08)	1.06(− 0.22,2.33)	3.04(0.88,5.20)	0.0944	< 0.001
Model 2	Ref	2.05(0.98,3.12)	1.42(−0.12,2.97)	2.49(− 0.19,5.17)	0.0778	< 0.001
Model 3	Ref	2.07(1.00,3.14)	1.46(−0.09,3.00)	2.49(−0.19,5.18)	0.0783	< 0.001
**DBP (mmHg)**	73.47 ± 11.22	74.59 ± 11.27	74.16 ± 11.28	75.83 ± 12.42		< 0.001
Crude model	Ref	1.12(0.65,1.59)	0.68(0.01,1.36)	2.35(1.22,3.49)	0.0012	< 0.001
Model 1	Ref	1.48(1.01,1.95)	0.78(0.11,1.45)	2.18(1.05,3.32)	0.0330	< 0.001
Model 2	Ref	1.26(0.69,1.82)	0.95(0.14,1.76)	2.00(0.60,3.41)	0.0306	< 0.001
Model 3	Ref	1.26(0.69,1.82)	0.99(0.18,1.79)	2.03(0.62,3.43)	0.0316	< 0.001
**Glucose (mmol/L)**	5.73 ± 1.63	5.85 ± 1.78	5.92 ± 1.85	6.03 ± 2.28		< 0.001
Crude model	Ref	0.118(0.048,0.117)	0.190(0.091,0.290)	0.299(0.130,0.468)	0.0010	< 0.001
Model 1	Ref	0.149(0.079,0.219)	0.259(0.158,0.359)	0.352(0.182,0.522)	0.0154	< 0.001
Model 2	Ref	0.155(0.069,0.240)	0.286(0.162,0.410)	0.330(0.113,0.546)	0.0176	< 0.001
Model 3	Ref	0.157(0.071,0.243)	0.279(0.155,0.403)	0,336(0.120,0.552)	0.0194	< 0.001
**HDL-cholesterol (mmol/L)**	1.66 ± 0.41	1.63 ± 0.40	1.63 ± 0.42	1.60 ± 0.40		< 0.001
Crude model	Ref	−0.029(−0.045,-0.012)	−0.030(− 0.055,-0.006)	− 0.060(− 0.101,-0.019)	0.0007	< 0.001
Model 1	Ref	− 0.032(− 0.049,-0.015)	− 0.017(− 0.041,0.007)	−0.029(− 0.070,0.012)	0.0494	0.001
Model 2	Ref	−0.020(− 0.040,0.000)	−0.015(− 0.044,0.014)	−0.020(− 0.070,0.031)	0.0498	0.054
Model 3	Ref	−0.018(− 0.038,0.002)	−0.019(− 0.048,0.010)	−0.026(− 0.076,0.024)	0.0560	0.030
**LDL-cholesterol (mmol/L)**	3.26 ± 0.71	3.26 ± 0.69	3.30 ± 0.71	3.31 ± 0.73		0.262
Crude model	Ref	−0.003(− 0.032,0.027)	0.034(− 0.008,0.076)	0.045(− 0.027,0.117)	0.0000	0.100
Model 1	Ref	−0.030(− 0.059,-0.001)	0.027(− 0.015,0.069)	0.064(− 0.007,0.135)	0.0483	0.335
Model 2	Ref	−0.009(− 0.043,0.025)	0.084(0.035,0.132)	0.043(− 0.042,0.128)	0.0610	0.012
Model 3	Ref	−0.006(− 0.039,0.028)	0.076(0.027,0.124)	0.033(− 0.051,0.118)	0.0689	0.023
**TG (mmol/L)**	1.66 ± 1.22	1.73 ± 1.34	1.83 ± 1.73	1.90 ± 1.61		< 0.001
Crude model	Ref	0.062(0.010,0.115)	0.166(0.091,0.242)	0.236(0.108,0.364)	0.0011	< 0.001
Model 1	Ref	0.067(0.014,0.121)	0.174(0.098,0.250)	0.242(0.113,0.372)	0.0029	< 0.001
Model 2	Ref	0.030(−0.032,0.093)	0.207(0.117,0.297)	0.224(0.067,0.381)	0.0036	< 0.001
Model 3	Ref	0.031(−0.031,0.093)	0.208(0.118,0.298)	0.227(0.070,0.384)	0.0038	< 0.001
**TC (mmol/L)**	5.92 ± 1.14	5.92 ± 1.13	5.95 ± 1.11	5.97 ± 1.17		0.764
Crude model	Ref	0.000(−0.048,0.048)	0.026(− 0.042,0.094)	0.047(− 0.069,0.162)	−0.0001	0.351
Model 1	Ref	−0.016(− 0.063,0.032)	0.045(− 0.023,0.113)	0.113(− 0.003,0.228)	0.0355	0.099
Model 2	Ref	−0.031(− 0.088,0.027)	0.062(− 0.021,0.145)	0.053(− 0.091,0.197)	0.0376	0.367
Model 3	Ref	−0.025(− 0.082,0.032)	0.050(− 0.033,0.132)	0.038(− 0.105,0.182)	0.0441	0.493

Data were expressed as β-coefficients and 95% confidence interval, or mean ± standard deviation

Model 1: adjusted for sex, age, education, household income, occupation, physical activity, drinking status, smoking status

Model 2: additionally adjusted for daily energy intake

Model 3: additionally adjusted for daily carbohydrate, fat and protein intake

Table 4 shows that, in participants without diabetes, positive associations of spicy food intake with DBP, HDL-cholesterol and adiposity measures were found. The associations became non-significant in participants with diabetes, except for BMI and WC, which showed similar positive associations.

**Table 4:** Associations of frequency and strength of spicy food consumption with CVD risk factors by diabetes

	**Frequency of spicy foods consumption; β and 95% confidence interval**					
	**Never(<1 days/wk.)**	**Sometimes (<2 days/wk.)**	**Often (3–4 days/wk.)**	**Usually (≥5 days/wk.)**	**Adjusted** ***R-*squared**	***P*** **for trend**
**No. of participants**						
Diabetes	3306	257	155	210		
Non-diabetes	22,821	1449	840	951		
**SBP**						
Diabetes	Ref	1.64(−1.52,4.80)	1.39(−2.63,5.41)	1.72(− 1.92,5.36)	0.0419	0.201
Non-diabetes	Ref	0.80(−0.55,2.16)	2.20(0.43,3.96)	2.27(0.58,3.95)	0.0789	< 0.001
**DBP**						
Diabetes	Ref	0.93(−0.68,2.53)	0.79(− 1.26,2.83)	1.98(0.13,3.82)	0.0277	0.022
Non-diabetes	Ref	0.67(− 0.05,1.38)	1.18(0.25,2.11)	1.85(0.96,2.74)	0.0334	< 0.001
**Glucose (mmol/L)**						
Diabetes	Ref	−0.047(− 0.497,0.403)	− 0.182(− 0.752,0.388)	0.440(− 0.073,0.952)	0.0103	0.297
Non-diabetes	Ref	0.010(− 0.029,0.049)	0.052(0.002,0.103)	0.026(− 0.023,0.075)	0.0707	0.054
**HDL-cholesterol (mmol/L)**						
Diabetes	Ref	−0.035(− 0.091,0.020)	0.002(− 0.069,0.073)	0.007(− 0.056,0.071)	0.0637	0.943
Non-diabetes	Ref	−0.004(− 0.030,0.021)	0.001(− 0.032,0.035)	−0.044(− 0.076,-0.012)	0.0572	0.027
**LDL-cholesterol (mmol/L)**						
Diabetes	Ref	0.021(−0.082,0.125)	−0.034(− 0.166,0.097)	0.007(− 0.111,0.125)	0.0641	0.972
Non-diabetes	Ref	0.067(0.025,0.110)	0.012(−0.043,0.067)	−0.047(− 0.099,0.006)	0.0716	0.732
**TG (mmol/L)**						
Diabetes	Ref	0.109(−0.156,0.375)	−0.011(− 0.348,0.327)	0.499(0.195,0.802)	0.0144	0.006
Non-diabetes	Ref	0.021(−0.050,0.092)	0.033(−0.059,0.125)	0.068(− 0.020,0.156)	0.0023	0.091
**TC (mmol/L)**						
Diabetes	Ref	−0.011(− 0.187,0.164)	−0.043(− 0.267,0.180)	0.089(− 0.112,0.290)	0.0364	0.612
Non-diabetes	Ref	0.068(−0.005,0.140)	−0.007(− 0.101,0.087)	−0.122(− 0.212,-0.032)	0.0471	0.092
**BMI (kg/m**^**2**^**)**						
Diabetes	Ref	0.71(0.25,1.18)	0.96(0.37,1.55)	1.16(0.63,1.69)	0.0221	< 0.001
Non-diabetes	Ref	0.55(0.34,0.76)	0.81(0.54,0.108)	1.11(0.85,1.37)	0.0215	< 0.001
**WC (cm)**						
Diabetes	Ref	1.11(−0.07,2.30)	2.05(0.54,3.56)	3.07(1.71,4.43)	0.0743	< 0.001
Non-diabetes	Ref	1.22(0.68,1.77)	1.69(0.98,2.40)	2.49(1.81,3.17)	0.0792	< 0.001
**WHR**						
Diabetes	Ref	0.001(−0.007,0.010)	0.008(− 0.003,0.019)	0.005(− 0.005,0.015)	0.1024	0.137
Non-diabetes	Ref	0.004(0.000,0.007)	0.007(0.002,0.012)	0.009(0.004,0.014)	0.1471	< 0.001
	**Strength of spicy foods consumption; β and 95% confidence interval**					
	**Never**	**Mild**	**Moderate**	**strong**	**Adjusted** ***R-*squared**	***P*** **for trend**
**No. of participants**						
Diabetes	3306	354	197	71		
Non-diabetes	22,821	2022	909	305		
**SBP**						
Diabetes	Ref	1.16(−1.63,3.96)	2.44(−1.14,6.01)	1.38 (−4.76,7.51)	0.0421	0.155
Non-diabetes	Ref	1.93(0.77,3.09)	0.58(−1.14,2.31)	2.34(−0.66,5.34)	0.0789	0.006
**DBP**						
Diabetes	Ref	1.04 (− 0.38,2.47)	1.61(− 0.20,3.42)	1.01(−2.11,4.13)	0.0275	0.043
Non-diabetes	Ref	1.23(0.62,1.84)	0.63(−0.28,1.54)	2.16(0.57,3.74)	0.0333	< 0.001
**Glucose (mmol/L)**						
Diabetes	Ref	0.068(−0.329,0.464)	0.032(−0.475,0.538)	0.260(− 0.619,1.138)	0.0093	0.595
Non-diabetes	Ref	0.044(0.010,0.077)	0.010(−0.040,0.059)	−0.052(− 0.139,0.035)	0.0708	0.466
**HDL-cholesterol (mmol/L)**						
Diabetes	Ref	−0.020(− 0.069,0.029)	0.000(− 0.063,0.063)	0.009(− 0.118,0.099)	0.0633	0.694
Non-diabetes	Ref	−0.014(− 0.036,0.007)	−0.013(− 0.045,0.019)	−0.014(− 0.071,0.042)	0.0569	0.173
**LDL-cholesterol (mmol/L)**						
Diabetes	Ref	−0.046(− 0.137,0.045)	0.102(− 0.014,0.218)	−0.044(− 0.244,0.156)	0.0655	0.610
Non-diabetes	Ref	0.001(−0.036,0.037)	0.056(0.002,0.110)	0.042(−0.053,0.136)	0.0711	0.071
**TG (mmol/L)**						
Diabetes	Ref	0.060(−0.175,0.294)	0.459(0.160,0.758)	0.184(−0.331,0.699)	0.0139	0.011
Non-diabetes	Ref	0.003(−0.057,0.064)	0.081(− 0.009,0.171)	0.164(0.007,0.321)	0.0025	0.020
**TC (mmol/L)**						
Diabetes	Ref	−0.040(− 0.195,0.115)	0.101(− 0.097,0.299)	0.023(− 0.318,0.364)	0.0366	0.590
Non-diabetes	Ref	−0.022(− 0.084,0.040)	0.016(− 0.076,0.108)	0.026(− 0.134,0.187)	0.0465	0.934
**BMI (kg/m**^**2**^**)**						
Diabetes	Ref	0.75(0.34,1.16)	0.99(0.47,1.51)	1.59(0.69,2.50)	0.0226	< 0.001
Non-diabetes	Ref	0.61(0.43,0.79)	1.02(0.76,1.29)	1.22(0.76,1.69)	0.0215	< 0.001
**WC (cm)**						
Diabetes	Ref	1.51(0.46,2.56)	1.99(0.66,3.33)	4.51(2.21,6.81)	0.0746	< 0.001
Non-diabetes	Ref	1.48(1.01,1.94)	1.83(1.14,2.52)	3.04(1.83,4.25)	0.0790	< 0.001
**WHR**						
Diabetes	Ref	0.003(−0.004,0.011)	0.007(−0.003,0.016)	0.001(− 0.016,0.018)	0.1022	0.214
Non-diabetes	Ref	0.006(0.002,0.009)	0.004(−0.001,0.009)	0.015(0.007,0.024)	0.1471	< 0.001

Data were expressed as β-coefficients and 95% confidence interval

## Discussion

Our study showed that higher frequency and strength of spicy foods intake were associated with unfavorable CVD risk profile, such as higher levels of BMI, WC, WHR, SBP, DBP, fasting plasma glucose and triglycerides, and lower levels of HDL-C. The positive associations of spicy foods intake with BMI, WC, triglycerides and DBP remained in diabetic patients. After adjusting for total energy, carbohydrate, fat and protein intakes, the associations between spicy food intake and cardiovascular risk factors remained, suggesting that energy and nutrients intake could not fully explain these associations. Since some potential confounders such as sweets intake and beverages were not measured and could not be controlled, further studies are needed to confirm these results. As spicy foods are very popular worldwide, our results provided evidence that light diet, rather than spicy foods might be more suitable for older people who had an elevated risk of CVD. Additionally, the association of spicy foods with cardiovascular disease risk factors may represent culture-mediated differences in the high energy density of spicy foods, which in cold weather regions are considered to be heat (or “yang”) and beneficial [29]. More work is needed to elucidate the mechanisms behind this finding. Furthermore, as the association may be modified by district or weather variabilities [30], further evidence from different settings is needed. Our findings may underlie a potential dietary recommendation for prevention of cardiovascular disease in older people, especially in regions with typically low consumption of spicy foods, such as southern China.

Results of the current study were consistent to some, but not all findings of other population-based studies. For example, ours as well as the CKB study showed positive associations of frequency and strength of spicy foods consumption with adiposity measures [31]. Moreover, the Rural Diabetes, Obesity and Lifestyle study also showed a positive association between spicy food preference and general obesity [20]. However, an inverse association was reported by the China Health and Nutrition Survey (CHNS) study, showing that chilli intake was associated with a lower risk of overweight and obesity [17]. A population-based study in Italy reported that chili pepper intake was associated with significantly lower risks of all-cause cardiovascular and cerebrovascular mortality. This study also showed significant associations of higher spicy foods intake with a more favorable cardiovascular and cerebrovascular disease risk profile including overweight and obesity [3]. Additionally, in our study, we found that higher intake of spicy foods was associated with higher levels of triglycerides. The association remained in those without diabetes. Besides, higher degree of pungency was associated with lower HDL-C concentrations, but the association became non-significant after adjustment for potential confounders. This result was consistent with the China Kadoorie Biobank Study in which spicy food was shown to be a risk factor for obesity and dyslipidemia [21]. Furthermore, we found that spicy foods consumption was also associated with higher blood pressure, especially DBP, which was inconsistent with the existing studies, in which spicy foods consumption was significantly associated with low blood pressure in women. One possible reason for this discrepancy is some of the studies with relatively small sample size did not control for total energy intake [32, 33].

In Chinese dishes, spices were often added to increase the palatability of dishes and stimulate people’s appetite. Besides, sweet food, beverage or alcohol were often consumed to cool the heat and pain caused by spicy foods which may contribute to the higher lipids or blood glucose level. In addition, in our population, spicy foods were often consumed in hotpots with excessive animal fats, which would increase sodium intake and lead to subsequently higher levels of blood pressure. A study showed that spicy food could elevate blood pressure transiently by diet-induced thermogenesis (DIT) [34]. However, information regarding the thermogenesis effects of spicy foods consumption was not collected and thus could not be taken into account in the current study. Furthermore, animal studies in an extremely strict experimental environment using exactly the same energy intake between subjects and controls showed that capsaicinoids [35] or green pepper juice reduced levels of triglycerides [36]. However, in real life, people consume spicy foods without a fixed total calories intake. The unfavorable effects of spicy foods on cardiovascular risk factors may reflect an unhealthy spicy-foods diet pattern rather than the capsaicinoids or pepper per se.

The strengths of this study include a large sample size, use of standardized data collection procedures, and comprehensively control for established and potential risk factors for cardiovascular disease. There were still some limitations in this study. First, causal inference could not be confirmed due to the observational nature of this study. Second, recall error was possible because information of frequency and strength of spicy foods intake was collected by self-reporting questionnaires, and the quantity and the recent recipe could not represent the long-time eating habits. More objective methods such as using food diary or blood biomarkers for nutrients can provide more accurate information, although they might not be feasible in large scale epidemiologic studies. The existence of recall error might lead to a less precise estimation. Third, as the number of diabetic patients was limited, estimates on those with diabetes had wide confidence interval. Further studies evaluating the association in a larger sample of diabetes patients are needed. Despite the limitations, the current study showed a real-world evaluation of the potential unfavorable effects of spicy foods intake on cardiovascular disease, which provided new perspective in metabolic disease control and prevention.

## Conclusions

In summary, the current large population-based cross-sectional study showed that more spicy foods intake, including both higher intake frequency and strength, was associated with unhealthy cardiovascular risk profile. The associations were independent of a wide range of potential confounders and in dose-response manner.

## Supplementary Information

**Additional file 1: Supplementary Figure 1.** Association of frequency with cardiovascular disease risk factors. **Supplementary Figure 2.** Association of pungency with cardiovascular disease risk factors.

## Data Availability

Due to privacy or ethical restrictions, the data that support the findings will be made available on requests from the Guangzhou Biobank Cohort Study Data Access Committee (gbcsdata@hku.hk). The datasets used and/or analyzed during the current study are available from the corresponding author on reasonable request.

## Abbreviations

CVD: Cardiovascular disease
BMI: Body mass index
CI: Confidence interval
WC: Waist circumference
WHR: Waist-to-hip ratio
SBP: Systolic blood pressure
DBP: Diastolic blood pressure
FPG: Fasting plasma glucose
TG: Triglycerides
HDL-C: Lower high density lipoprotein cholesterol
TC: Total cholesterol
LDL-C: Low density lipoprotein cholesterol
GBCS: The Guangzhou Biobank Cohort Study
CHNS: China Health and Nutrition Survey
