# Supplementary material for: Association between spicy foods consumption and cardiovascular disease risk factors: Guangzhou Biobank Cohort Study
Source: BMC Public Health. 2022 Jun 30;22:1278. doi: 10.1186/s12889-022-13697-6 (PMC9248154; doi:10.1186/s12889-022-13697-6)
Supplement: Supplementary file 1 — Additional file 1: Supplementary Figure 1. Association of frequency with cardiovascular disease risk factors. Supplementary Figure 2. Association of pungency with cardiovascular disease risk factors. [file 12889_2022_13697_MOESM1_ESM.docx]

Supplementary Figure 1. Association of frequency with cardiovascular disease risk factors.

The panels show association between (a) body mass index, (b) waist circumference, (c) diastolic blood pressure, (d) fasting plasma glucose, (e) HDL-cholesterol, (f) triglyceride and frequency of spicy food consumption respectively.

The gray area indicates 95% prediction interval.

Supplementary Figure 2. Association of pungency with cardiovascular disease risk factors.

The panels show association between (a) body mass index, (b) waist circumference, (c) diastolic blood pressure, (d) fasting plasma glucose, (e) HDL-cholesterol, (f) triglyceride and pungency of spicy food consumption respectively.

The gray area indicates 95% prediction interval.
